# Impact of the Duration of Trimethoprim–Sulfamethoxazole Prophylaxis on the Incidence of Infection After Kidney Transplantation: A Target Trial Emulation Study Within the Swiss Transplant Cohort Study (STCS)—The QUID‐PRO‐QUO Study (QUIDney Transplantation and Duration of PROphylaxis With QUO‐Trimoxazole)

**DOI:** 10.1111/tid.70106

**Published:** 2025-09-18

**Authors:** Aline Munting, Frédérique Chammartin, Isabelle Binet, Katia Boggian, Michael Dickenmann, Marc Froissart, Christian Garzoni, Dela Golshayan, Fadi Haidar, Cédric Hirzel, Kerstin Hübel, Uyen Huynh‐Do, Nina Khanna, Michael Koller, Nicolas Mueller, Daniel Sidler, Christian van Delden, Oriol Manuel, Swiss Transplant Cohort Study

**Affiliations:** ^1^ Infectious Diseases Service,University Hospital of Lausanne and University of Lausanne Lausanne Switzerland; ^2^ Department of Clinical Research Division of Clinical Epidemiology University Hospital Basel University of Basel Basel Switzerland; ^3^ Clinic of Nephrology and Transplantation Medicine HOCH Health Ostschweiz Cantonal Hospital St. Gallen St. Gallen Switzerland; ^4^ Division of Infectious Diseases Infection Prevention and Travel Medicine HOCH Health Ostschweiz Kantonsspital St. Gallen University Teaching and Research Hospital St. Gallen Switzerland; ^5^ Clinic for Transplantation Immunology and Nephrology University Hospital Basel Basel Switzerland; ^6^ Clinical Trial Unit Lausanne University Hospital and University of Lausanne Lausanne Switzerland; ^7^ Department of Infectious Diseases Inseslspital Bern University Hospital University of Bern Bern Switzerland; ^8^ Clinic of Internal Medicine and Infectious Diseases Clinica Luganese Lugano Switzerland; ^9^ Transplantation Center Lausanne University Hospital and University of Lausanne Lausanne Switzerland; ^10^ Department of Medicine Division of Nephrology University Hospital of Geneva Geneva Switzerland; ^11^ Clinic of Nephrology University Hospital Zurich Zurich Switzerland; ^12^ Division of Nephrology and Hypertension University Hospital Bern Bern Switzerland; ^13^ Infectious Diseases and Hospital Epidemiology University Hospital Basel Basel Switzerland; ^14^ Department of Infectious Diseases and Hospital Epidemiology University Hospital Zurich University of Zurich Zurich Switzerland; ^15^ Transplant Infectious Diseases Unit University Hospitals Geneva and Faculty of Medicine Geneva Switzerland

## Abstract

**Background:**

Trimethoprim–sulfamethoxazole prophylaxis effectively prevents opportunistic and non‐opportunistic infections in kidney transplantation, but optimal duration remains uncertain. This study investigated whether extending TMP–SMX prophylaxis is associated with lower infection rates.

**Methods:**

This target trial emulation using observational data from the Swiss Transplant Cohort Study compared short (< 7 months) versus long (≥ 7 months) TMP–SMX prophylaxis. The primary outcome was bacterial infection potentially susceptible to TMP–SMX up to 12‐months post‐transplant. Inverse probability weighting (IPW) adjusted for confounders including age, living donation, lymphocyte counts, use of antithymocyte globulin, acute rejection, CMV infection, and transplant center. All bacterial and opportunistic infections, kidney function, and patient and allograft survival were summarized descriptively.

**Results:**

A total of 1700 KTRs fulfilled inclusion criteria; 1325 (78%) participants received a short prophylaxis and 375 (22%) received a long prophylaxis. Median TMP–SMX duration was 179 days in the short group and 280 days in the long group. At 12‐month post‐transplant, the primary outcome was observed in 120/1325 (9.1%) in the short group and 43/375 (11.5%) in the long group. IPW analysis estimated an adjusted risk difference of 2.11% (95% CI −0.47% to 5.27%). Center, rejection, and use of ATG were associated with longer TMP–SMX duration, but risk difference was similar before and after weighting. Urinary tract infection was the most common bacterial infection. Opportunistic and overall infection rates, kidney function, and patient and graft survival were similar among groups.

**Conclusions:**

In this target trial emulation, no differences in bacterial infection rates at 12‐month post‐transplant was observed between short and long TMP–SMX prophylaxis.

**Abbreviations**
ALC: absolute lymphocytes count
ATG: anti‐thymocyte globulin
CI: confidence interval
CMV: cytomegalovirus
IPW: inverse probability weighting
IQR: interquartile range
KTRs: kidney transplant recipients
PCP: *Pneumocystis pneumonia*
PY: person‐year
SOT: solid organ transplantation
STCS: Swiss Transplant Cohort Study
TMP–SMX: trimethoprim–sulfamethoxazole
UTI: urinary tract infections

## 1 Introduction

Solid organ transplantation (SOT) recipients face heightened infection risks. Trimethoprim–sulfamethoxazole (TMP–SMX) prophylaxis is widely used after transplantation for the prevention of opportunistic infections, mainly *Pneumocystis* pneumonia (PCP), and has led to a reduction incidence from 5%–15% to

0.3%–3% in recent series [1, 2, 3]. There are several other advantages of the use of TMP–SMX prophylaxis including low cost, relatively low toxicity, ease of administration, and antimicrobial activity against a broad spectrum of pathogens, such as *Toxoplasma, Nocardia*, and *Listeria* [4]. TMP–SMX has also antibacterial activity against many bacterial pathogens, such as staphylococci, streptococci, Enterobacteriaceae and some non‐fermentative Gram‐negative bacteria. Infections from these pathogens are very frequent throughout the first‐year post‐transplant and may account for more than 60% of all infections in kidney transplant recipients (KTRs) [5].

TMP–SMX prophylaxis in KTRs is generally started early after transplant for a total duration varying from 6 to 12 months, with some patients receiving longer durations depending on the local guidelines, type of induction, maintenance immunosuppressive therapy, and previous episodes of rejection [6, 7, 8, 9].

There are scarce data assessing whether duration of TMP–SMX for more than 6 months in KTRs is associated with lower incidence of infections, including both opportunistic and “classical” bacterial infections, the latter being much more common and potentially life‐threatening. Therefore, we took the advantage of the large number of patients included in Swiss Transplant Cohort Study (STCS) to assess the impact of two different durations of TMP–SMX prophylaxis on bacterial and opportunistic infections in KTRs, hypothesizing that patients who receive longer durations of TMP–SMX would develop lower rates of bacterial infections. Given the potential prescription bias of longer duration of TMP–SMX prophylaxis in patients at higher risk of infection, we aimed to emulate a target trial by weighting our study population to balance individuals’ characteristics and mitigate confounding.

## 2 Methods

### 2.1 Study Design

We emulated a target trial that aimed to compare “short” and “long” duration of TMP–SMX prophylaxis in KTRs with regards to bacterial infection. We used observational data from the STCS database, an ongoing prospective cohort established in 2008, that includes more than 90% of SOT recipients in Switzerland. All centers’ local ethical committees approved the cohort study, and all patients provided written informed consent. The Ethics Committee of the Vaud Canton (CER‐VD) approved this study (2022‐00192).

### 2.2 Eligibility Criteria

We considered all KTRs participating in the STCS since May 2008 until December 2022 with at least 7 months of follow‐up. All patients receiving TMP–SMX prophylaxis at 5 months post‐transplantation were included in the “short prophylaxis” group if they were no longer under TMP–SMX prophylaxis at 7 months post‐transplantation and in the “long prophylaxis” group if they still had TMP–SMX prophylaxis at 7 months post‐transplantation. We excluded patients with less than 5 months of prophylaxis, as the standard practice in the STCS is 6 months of prophylaxis and such patients might not have been comparable. We only considered adults participants who had started prophylaxis within the first 2 months post‐transplant and were alive and with functioning allograft at 7 months post‐transplant. In case of retransplantation, we only considered the most recent procedure. Exclusion criteria were the lack of consent form, and patients with previous or concomitant non‐kidney transplantation.

### 2.3 Outcomes, Follow‐Up, and Additional Definitions

Time 0 for the target trial was defined at 7‐month post‐transplantation, where participants were defined as part of the long or short antibiotic prophylaxis group. The primary outcome was a proven infection potentially susceptible to TMP–SMX at 5‐month follow‐up from Time 0 (12‐month post‐transplant). Potentially TMP–SMX‐susceptible bacterial infections (thereafter named potentially susceptible bacterial infection) included staphylococci, streptococci, *Haemophilus*, Enterobacteriaceae (*Escherichia coli, Klebsiella, Proteus, Enterobacter, Morganella*)*, Stenotrophomonas* spp*., and Acinetobacter spp*. Confirmed susceptibility to TMP–SMX was not available since antimicrobial susceptibility pattern is not routinely collected in the STCS (the STCS collects essentially whether the bacterial pathogen is multidrug resistant including extended‐spectrum beta‐lactamase‐ and carbapenemase‐producers). The definition of infection followed the STCS Infectious Diseases guidelines for the diagnosis of proven bacterial infection (i.e. isolated pathogen with clinical signs and/or symptoms and treatment given) [5].

Secondary outcomes were 1) potentially susceptible bacterial infections at 17‐month follow‐up (24 months post‐transplant), 2) all bacterial infections at 17‐month follow‐up, and 3) opportunistic infections at 17‐month follow‐up. Opportunistic infections included PCP, toxoplasmosis, and nocardiosis. We also described breakthrough infections that occurred in the long prophylaxis group during TMP–SMX prophylaxis. Safety endpoints were kidney function, episodes of agranulocytosis, allograft, and patient survival at 5‐ and 17‐month follow‐up.

Participants were followed from Time 0 until the first event of infections, loss to follow‐up, graft loss, deaths, last cohort visit, or database closure, whatever comes first. Duration of prophylaxis was defined as the total duration, counting every day, for which the patients have been under prophylaxis, allowing interruptions of a maximum of 4 weeks. Prophylaxis was administered as a daily single‐strength regimen in two centers and as a double‐strength tablet three times per week in four centers.

### 2.4 Data Collection

Clinical variables used for the inverse probability weighting (IPW) included age, living donation, transplantation center, absolute lymphocytes count (ALC), use of anti‐thymocyte globulin (ATG), acute rejection (proven and clinically suspected according to STCS definition [10], and cytomegalovirus (CMV) infection within 7‐month post‐transplant, defined as viral replication irrespective of attributable symptoms. In addition, we collected available data regarding demographic and clinical data of study population, occurrence of infections, as well as patient and graft survival, induction and maintenance immunosuppressive therapy, diagnosis, and treatment of rejection. ALC are not routinely collected in STCS and were extracted from hospital electronic files.

### 2.5 Statistical Analysis

Prophylaxis duration is prone to selection bias in observational data and traditional statistics might bias estimates of the intervention effect. Therefore, we used the target trial emulation framework to emulate a target trial by weighting our study population to reduce imbalance between treatment arms and allow a fair comparison of bacterial infections between individuals receiving a short versus a long duration of TMP–SMX prophylaxis. This statistical approach has proved to provide results that are comparable to results obtained from a randomized study, under assumptions of correct model specification (no unmeasured confounding and no informative censoring), positivity, and consistency [11]. Stabilized treatment weights were calculated by fitting a logistic regression model with duration of prophylaxis as the outcome (short vs. long prophylaxis), and covariates: age at transplantation in years, living donor (no/yes), use of ATG (no/yes), prior biopsy proven or clinically suspected rejection (no/yes), CMV infection (no/yes), lymphocyte counts and study center. Those covariates were chosen by expert clinicians according to their confounding potential due to their assumed influence on decisions linked to a prolongation of an antibiotic prophylaxis. We incorporated the estimated inverse probability weights into a pooled logistic regression model where the occurrence of proven infections potentially susceptible to TMP–SMX was modelled with the covariates mentioned above. Follow‐up time in weeks was included in the model using a cubic polynomial spline. We estimated marginal probabilities of bacterial infection up to 24‐month post‐transplant, and calculated risk differences and risk ratios between both groups at 5‐ and 17‐month after weighting. We calculated 95% confidence interval (CI) using 200 nonparametric bootstrap samples. We analyzed our data according to an intention‐to‐treat principle where we included in the analysis of the long prophylaxis group all individuals that were on antibiotic prophylaxis at Month 7, irrespectively of the final duration of prophylaxis. All analyses were performed with R version 4.3.3.

## 3 Results

### 3.1 Study Population

Out of the 4135 KTRs enrolled in STCS, 1700 satisfied inclusion criteria (Figure S1). Most participants were excluded due to absence of TMP–SMX prophylaxis at 5 months or no measured ALC. A total of 1325 out of 1700 (78%) participants were assigned to the short prophylaxis group, and 375 out of 1700 (22%) to the long prophylaxis group. At 7‐month post‐transplant (time of the IPW), the median age was 55 years old (interquartile range [IQR], 45–64 years), and 35.9% (610/1700) were women. Percentages of maintenance immunosuppression regimen, use of ATG, and previous CMV infections were comparable in both groups. The proportion of individuals with a long prophylaxis varied across centers, suggesting local preferences with regards to prescription habits. Median TMP–SMX duration was 179 days (IQR 171–187) in the short prophylaxis group and 280 days (IQR 230–377) in the long prophylaxis group (Table 1). In the long prophylaxis group, 260 patients had 7–12 months of prophylaxis and 92 was still on prophylaxis between 12 and 24 months post‐transplant. Median duration of follow‐up was 16.9 months from 7‐month post‐transplant.

**TABLE 1:** Baseline characteristics of participants according to antibiotic prophylaxis duration at 7‐month post‐transplant.

	**Short prophylaxis group** **(*n* = 1325)**	**Long prophylaxis group** **(*n* = 375)**	**Total** **(*n* = 1700)**
**Prophylaxis duration in days, median (IQR)**	179 (171–187)	280 (230–377)	183 (174–202)
**Age, median (IQR), y**	55 (44,64)	56 (47,64)	55 (45,64)
**Living donor, n (%)**	551 (41.6)	138 (36.8)	689 (40.5)
**Female, n (%)**	477 (36)	133 (35.5)	610 (35.9)
**Centers, n (%)**			
Center 1	50 (3.8)	88 (23.5)	138 (8.1)
Center 2	295 (22.3)	71 (18.9)	366 (21.5)
Center 3	17 (1.3)	10 (2.7)	27 (1.6)
Center 4	98 (7.4)	14 (3.7)	112 (6.6)
Center 5	323 (24.4)	102 (27.2)	425 (25.0)
Center 6	542 (40.9)	90 (24.0)	632 (37.2)
**Induction therapy**, n (%)			
Polyclonal lymphocyte depleting antibodies (ATG)	347 (26.2)	107 (28.5)	454 (26.7)
Basiliximab	941 (71.0)	274 (73.1)	1215 (71.5)
Rituximab	84 (6.3)	28 (7.5)	112 (6.6)
Intravenous immunoglobulins	86 (6.5)	31 (8.3)	117 (6.9)
Other	37 (2.8)	11 (2.9)	48 (2.8)
**ABO incompatible transplantation, n (%)**	81 (6.1)	30 (8.0)	111 (6.5)
**Previous kidney transplant, n (%)**	194 (14.6)	60 (16.0)	254 (14.9)
**Maintenance therapy**, **n (%)**			
Prednisone	1282 (96.8)	358 (95.5)	1640 (96.5)
Tacrolimus	1194 (90.1)	290 (77.3)	1484 (87.3)
Mycophenolate	1287 (97.1)	367 (97.9)	1654 (97.3)
Cyclosporine	161 (12.2)	106 (28.3)	267 (15.7)
Azathioprine	75 (5.7)	23 (6.1)	98 (5.8)
**CMV infection between Tx and 7 month‐post Tx**, **n (%)**	370 (27.9)	140 (37.3)	510 (30)
**Rejection**, **n (%)**	306 (23.1)	143 (38.1)	449 (26.4)
**Total lymphocytes cell count, median (IQR)**	1210 (795,1720)	1120 (712, 1572)	1190 (770, 1700)

Abbreviations: CMV: cytomegalovirus, IQR: interquartile range

### 3.2 Primary Outcome

**FIGURE 1:**
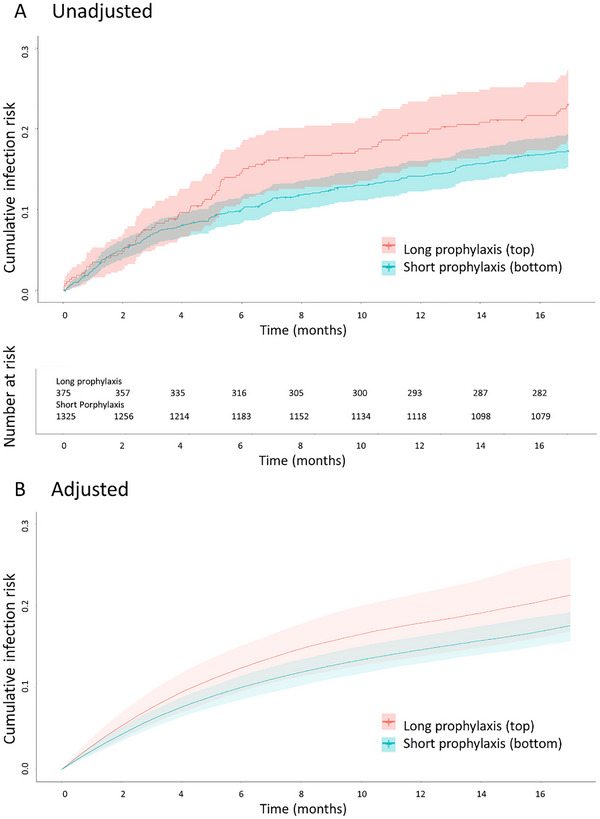
Cumulative incidence of infection potentially susceptible to trimethoprim–sulfamethoxazole. (A) The curves represent the unadjusted cumulative incidence of infection potentially susceptible to trimethoprim–sulfamethoxazole over time. (B) Curves of cumulative incidence of infection potentially susceptible to trimethoprim–sulfamethoxazole over time, adjusted for confounders (see Section 2 for a full list of covariates). Shaded areas represent 95% confidence intervals. Time 0 indicates the start of follow‐up and corresponds to 7‐month post‐transplant.

**FIGURE 2:**
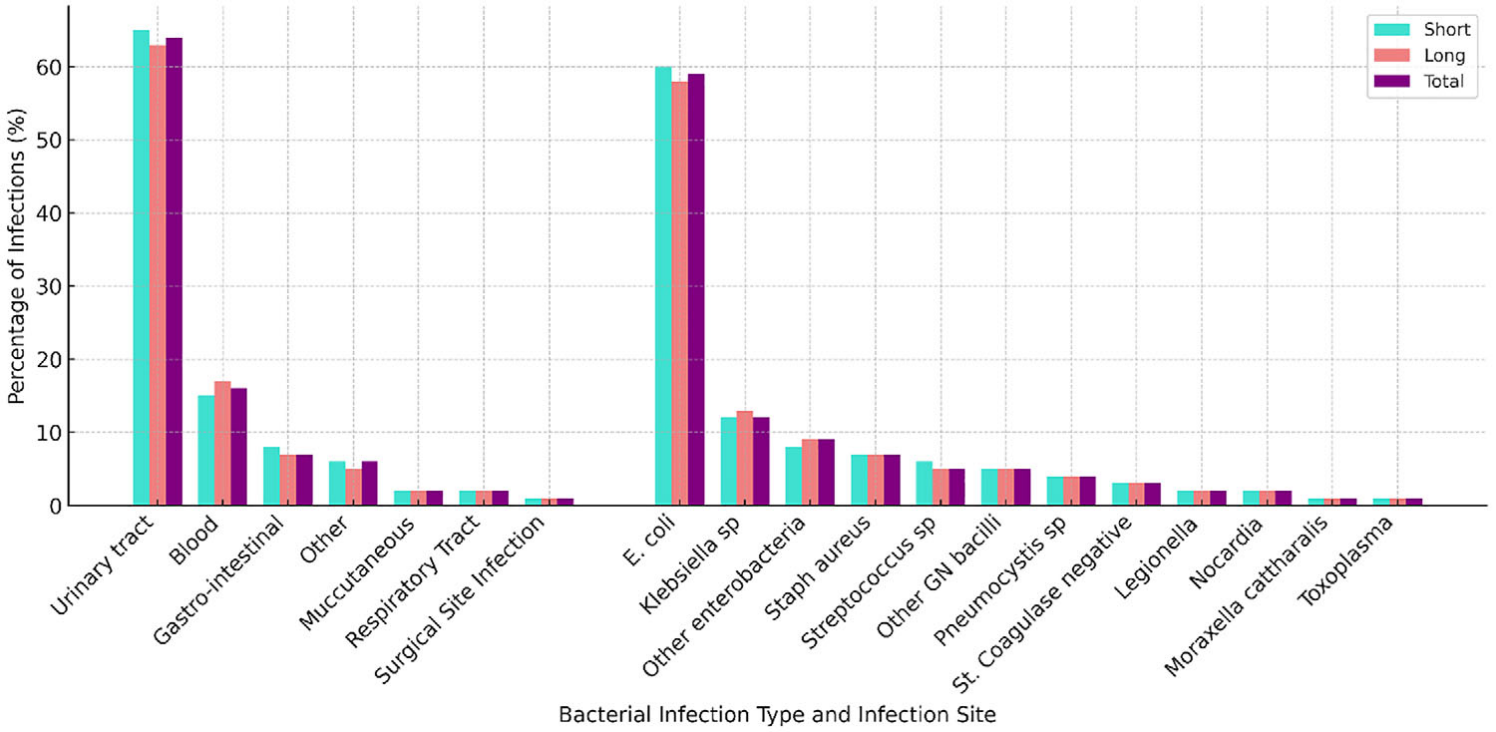
Type of infection (A) and pathogen (B) distribution at 17‐ month follow‐up, overall, and by prophylaxis group.

At 5‐month follow‐up (12‐month post‐transplant), 163/1700 (9.5%) of participants developed a potentially susceptible bacterial infection; 120/1325 (9.1%) In the short and 43/135 (11.5%) in the long prophylaxis groups. The overall incidence rate was 0.4 (95% CI: 0.3–0.4) per 1000 person‐years (PY) in the short prophylaxis group and 0.5 (95% CI: 0.4–0.7) per 1000 PY in the long prophylaxis group. Figure 1A shows the cumulative incidence curve with number at risks over 17‐month follow‐up. Unadjusted cumulative incidence of infections was 9.2% (95% CI: 7.8–10.6) in the short prophylaxis group and 11.2% (95% CI: 8.7–14.0) in the long prophylaxis group. Median time to onset of infection was 52 days (IQR: 29–89) in the short prophylaxis group and 74 days (IQR: 26–106) in long prophylaxis group. Overall, most infections were urinary tract infections (UTI) due to *Enterobacterales*, particularly *E. coli* (Figure 2). Approximately, 8.5% of ESBL producing bacteria were found in the short prophylaxis group and 8.6% in the long prophylaxis group.

Center, prior episode of rejection, use of ATG, and CMV infection within 7‐months post‐transplant were variables associated with longer prescriptions of TMP–SMX prophylaxis in the model to calculate weights (*p* value < 0.05) (Table S1). Contrary to our initial assumption, ALC were not statistically associated to the probability of being in the long antibiotic prophylaxis group (*p* value = 0.94). However, duration of prophylaxis was independently associated with centers, reflecting different practices among clinicians.

**FIGURE 3:**
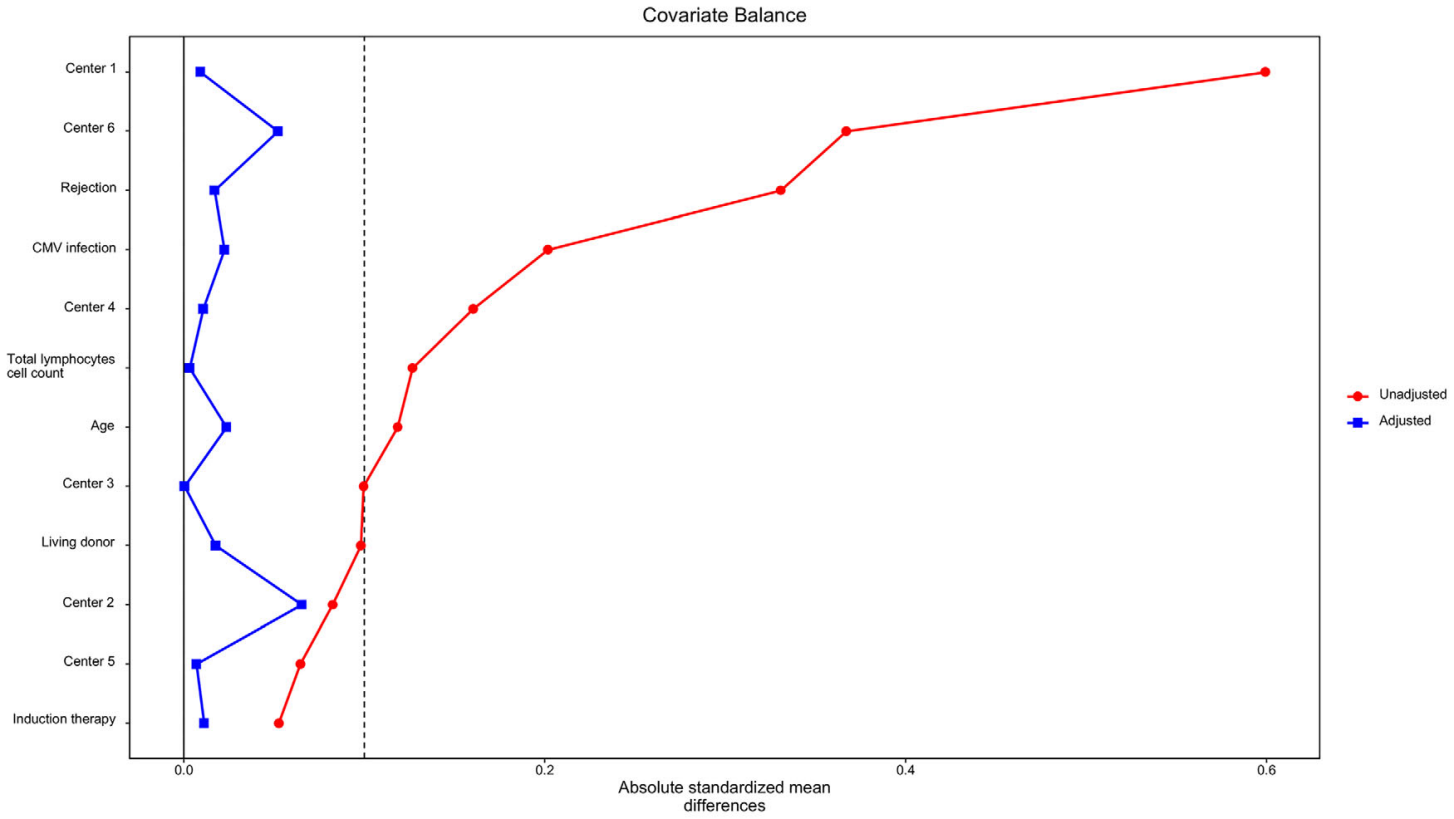
Covariate balance before (red) and after (blue) inverse probability weighting. The plot shows the absolute standardized mean difference between covariates in the unadjusted (red) and in the inverse probability weighted population (blue). They vertical grey line indicates a SDM of 0.1. Covariates below this line are considered to be well‐balanced between the two groups.

After adjusting, using the IPW model, covariates were considered well‐balanced between the two groups (Figure 3). Adjusted cumulative incidence was 9% (95% CI: 7.6–10.2) in the short prophylaxis group and 11.1% (95% CI: 8.4–13.7) in the long prophylaxis group (risk difference of 2.1% [95% CI: −0.5; 5.2]) (Figure 1B and Table 2). Age, use of ATG, and previous CMV infection were significantly associated with an increased risk of bacterial infection, whereas duration of prophylaxis was not (Table S2). Renal function was comparable between groups (Table S3), and the incidence of agranulocytosis was low and similar (0.5% in the short‐duration group vs. 0.3% in the long‐duration group).

**TABLE 2:** Bacterial infection risks in short and long antibiotic prophylaxis group, adjusted risk difference and adjusted risk ratio at 5‐ and 17‐month follow‐up. Adjusted results are adjusted for baseline confounders (see Section 2).

	**Short prophylaxis**		**Long prophylaxis**			
	Unadjusted risk (%)	Adjusted risk^a^ (%)	Unadjusted risk (%)	Adjusted risk^a^ (%)	**Adjusted risk** **difference** ^a^ **(%)**	**Adjusted risk** **ratio** ^a^
**5‐month follow‐up**	9.2 (7.8; 10.6)	9.0 (7.6; 10.2)	11.2 (8.7; 14.0)	11.1 (8.4; 13.7)	2.1 (‐0.5; 5.3)	1.23 (1.0; 1.6)
**17‐month follow‐up**	17.6 (15.8; 19.5)	17.6 (15.7; 19.2)	21.2 (16.9; 25.7)	21.3 (16.9; 25.9)	3.8 (‐0.8; 9.5)	1.21 (1.0; 1.6)

^a^ Adjusted for confounding (see Section 2 for a detailed list of covariates)

### 3.3 Secondary Outcomes

At 17‐month follow‐up (24‐month post‐transplant), adjusted cumulative incidence of potentially susceptible bacterial infections was 17.6% (95% CI: 15.7–19.2) in the short prophylaxis group and 21.3% (95% CI: 16.9–25.9) in the long prophylaxis group (adjusted risk difference 3.8 % [95%CI: −0.8 to 9.5]) (Figure 1B and Table 2). Incidence rate of all bacterial infection was 0.5 (95% CI: 0.5–0.6) and 0.7 (95% CI: 0.6–0.8) per 1000 PY in the short and long prophylaxis group, respectively. Incidence rate for opportunistic infections was 0.01 (95% CI: 0.01–0.02) per 1000 PY in the short antibiotic prophylaxis group and 0.03 (95% CI: 0.01–0.06) per 1000 PY in the long antibiotic prophylaxis group. Opportunistic pathogens are described in the Table S4. Breakthrough infections at 17‐month follow‐up occurred in 36 individuals in the long prophylaxis group, and were mostly due to *E. coli* and *Klebsiella* spp.

At the 17‐month follow‐up, there were 12 (0.7%) graft losses and 5 (0.3%) dropouts. A total of 21 (1.2%) deaths were recorded, with 12 out of 1 325 (0.9%) occurring in the short prophylaxis group and 9 out of 375 (2.4%) in the long prophylaxis group. Kidney function, graft rejection and survival at 5‐ and 17‐month follow‐up were comparable in the short and long prophylaxis groups (Table S3).

## 4 Discussion

This nationwide cohort study found no evidence of lower infection rates among KTRs receiving extended TMP–SMX prophylaxis compared to those on a standard duration of 6 months.

Several factors may explain these findings. First, despite using IPW to adjust for potential prescription bias, we cannot exclude some residual confounding for indication, as most centers extended prophylaxis beyond 6 months in patients undergoing treatment for rejection. Use of ATG, CMV infection, allograft rejection, and ALC were use in our model for the weighting. We included ALC in our model as a proxy of CD4 cell counts, which were not routinely measured in most participants. We found no association between low lymphocyte count and the probability of being in the long prophylaxis group, likely due to different practices across centers.

Second, most infections encountered were UTI caused by *Enterobacterales*, with a very low incidence of opportunistic infections in both groups. This suggests that longer duration of TMP–SMX prophylaxis did not effectively protect against common bacterial infections, likely due to the emergence of TMP–SMX resistant bacteria or due to suboptimal dosing (as seen for the prevention of nocardiosis) [12]). Of note, in Switzerland, *Enterobacterales* resistant to TMP–SMX ranges from 1% to up to 27%, being 23% for *E. coli*, 12% for *Klebsiella pneumonia*, and 6% for *Enterobacter*, although data specifically in SOT recipients in not available [13]. In our cohort, we did not find any difference on incidence of ESBL‐producing bacteria between the two groups.

Extended TMP–SMX prophylaxis in KTRs is proposed by some guidelines to prevent opportunistic infections in patients with enhanced immunosuppression. This includes patients undergoing anti‐rejection therapy, those with recurrent or protracted CMV infection, prolonged corticosteroid therapy (e.g., >20 mg daily), prolonged neutropenia, or flares of autoimmune disease [9]). However, this approach is based on limited evidence. Published results are controversial regarding the effect of TMP–SMX prophylaxis on preventing bacterial infections. Some retrospective cohort studies found that TMP–SMX was associated with a decreased risk of UTI [14, 15, 16], but two of them included asymptomatic bacteriuria in their definition of UTI. Another observational study from the STCS also demonstrated a reduction of bacteriemia during the first‐year post‐transplant with the use of TMP–SMX prophylaxis [17]. Furthermore, a meta‐analysis involving non‐HIV immunocompromised patients showed an advantage for TMP–SMX prophylaxis compared to no treatment in preventing bacterial infections (RR 0.4, 95% CI: 0.21–0.9), although data were mostly observational, the study population was heterogeneous, and no SOT recipients were included in the analysis [7]. One retrospective study analyzing the impact of TMP–SMX on proven UTI did not find any difference between 3 or 6 months of TMP–SMX prophylaxis in KTRs [2].

Regarding PCP, the highest risk is often reported within the first 6 months post‐transplantation, but factors such as treatment for acute rejection could prolong this risk. We recorded 18 cases of PCP during follow‐up, 11 in the short prophylaxis group, and 7 in the long prophylaxis group, all after discontinuation of TMP–SMX. Retrospective studies observed a median time of PCP diagnosis around 2 years after transplantation, frequently presenting in clusters suggesting inter‐human transmission [2, 18]. These data highlight that TMP–SMX prophylaxis is highly effective in preventing PCP. However, given the low overall incidence, extending prophylaxis beyond 6 months should be reserved for selected cases, including patients receiving ATG for acute rejection therapy or during an unexpected cluster of PCP cases within a transplant center [1, 8, 19].

Our study has several limitations. First and more obvious, this was an observational study prone to prescription biases. Although we accounted for main known selection biases, there may be residual unmeasured confounding, highlighted by the observed unexpected higher rate of infections in the long prophylaxis group. Second, as CD4 cell counts were not systematically measured at all centers at the time of stopping prophylaxis, we were not able to use CD4 cell count in our model – a known risk factor for PCP [20]; however, it seems unlikely that CD4 levels influenced the decision to prescribe extended prophylaxis, as discontinuation of prophylaxis was not based on CD4 cell counts in clinical practice. Third, only 27% of participants in the long prophylaxis group received up to 12 months of TMP–SMX, with a median of 9 months post‐transplant, reflecting real‐life practices. Comparing directly 6 versus 12 months would have increased the risk of bias by excluding fragile individuals experiencing drug toxicity, allograft lost and death. We addressed this issue by analyzing by intention‐to‐treat as in a clinical trial. Finally, we could not assess the impact of TMP–SMX resistance on the incidence of bacterial infection, given the potential high risk of TMP–SMX resistant pathogens in SOT recipients, widely exposed to TMP–SMX. In addition, our cohort database did not contain sufficiently detailed information to reliably identify drug‐related toxicities specifically attributable to TMP–SXT, but data on agranulocytosis and kidney function were similar between groups.

In conclusion, in this target trial emulation study in KTRs, we did not find differences in bacterial infection rates between a short and a long duration of TMP–SMX prophylaxis. Despite potential residual confounding, our findings do not support a systematic extension of the duration of TMP–SMX prophylaxis beyond 6 months after kidney transplantation.

## Conflicts of Interest

The authors have no conflicts of interest to disclose.

## Members of the Swiss Transplant Cohort Study

Patrizia Amico, Adrian Bachofner, Vanessa Banz, Sonja Beckmann, Guido Beldi, Christoph Berger, Ekaterine Berishvili, Annalisa Berzigotti, Françoise‐Isabelle Binet, Pierre‐Yves Bochud, Petra Borner, Sanda Branca, Anne Cairoli, Emmanuelle Catana, Yves Chalandon, Philippe Compagnon, Sabina De Geest, Sophie De Seigneux, Michael Dickenmann, Joëlle Lynn Dreifuss, Thomas Fehr, Sylvie Ferrari‐Lacraz, Andreas Flammer, Jaromil Frossard, Déla Golshayan, Nicolas Goossens, Fadi Haidar, Jürg Halter, Christoph Hess, Sven Hillinger, Hans Hirsch, Patricia Hirt, Linard Hoessly, Uyen Huynh‐Do, Franz Immer, Nina Khanna, Michael Koller, Angela Koutsokera, Andreas Kremer, Thorsten Krueger, Christian Kuhn, Arnaud L'Huillier, Bettina Laesser, Frédéric Lamoth, Roger Lehmann, Alexander Leichtle, Oriol Manuel, Hans‐Peter Marti, Michele Martinelli, Valérie McLin, Katell Mellac, Aurélia Merçay, Karin Mettler, Sara Christina Meyer, Nicolas Müller, Jelena Müller, Ulrike Müller‐Arndt, Mirjam Nägeli, Dionysios Neofytos, Jakob Nilsson, Manuel Pascual, Rosmarie Pazeller, David Reineke, Juliane Rick, Fabian Rössler, Silvia Rothlin, Thomas Schachtner, Stefan Schaub, Dominik Schneidawind, Macé Schuurmans, Simon Schwab, Thierry Sengstag, Daniel Sidler, Federico Simonetta, Jürg Steiger, Guido Stirnimann, Ueli Stürzinger, Christian Van Delden, Jean‐Pierre Venetz, Jean Villard, Julien Vionnet, Laura Walti, Caroline Wehmeier, Patrick Yerly.

## Supporting information




**Supplementary Table 1**: Logistic regression model parameters to estimate the probability of a long antibiotic prophylaxis to derive inverse probability weights.
**Supplementary Table 2**: Logistic regression model parameters of IPW model for the risk of bacterial infection.
**Supplementary table 3**: Safety outcomes at 17‐month follow‐up by prophylaxis group.
**Supplementary table 4**: Opportunistic infection (based on first episode), overall and by prophylaxis group.

## Data Availability

The data underlying this study were obtained from the Swiss Transplant Cohort Study (STCS). The data that support the findings of this study are available on request from the corresponding author. The data are not publicly available due to privacy or ethical restrictions.
