# Supplementary material for: Impact of the Duration of Trimethoprim–Sulfamethoxazole Prophylaxis on the Incidence of Infection After Kidney Transplantation: A Target Trial Emulation Study Within the Swiss Transplant Cohort Study (STCS)—The QUID‐PRO‐QUO Study (QUIDney Transplantation and Duration of PROphylaxis With QUO‐Trimoxazole)
Source: Transpl Infect Dis. 2025 Sep 18;27(5):e70106. doi: 10.1111/tid.70106 (PMC12519920; doi:10.1111/tid.70106)
Supplement: Supplementary file 1 — Supplementary Table 1: Logistic regression model parameters to estimate the probability of a long antibiotic prophylaxis to derive inverse probability weights. Supplementary Table 2: Logistic regression model parameters of IPW model for the risk of bacterial infection. Supplementary table 3: Safety outcomes at 17‐month follow‐up by prophylaxis group. Supplementary table 4: Opportunistic infection (based on first episode), overall and by prophylaxis group. [file TID-27-e70106-s001.docx]

**Supplementary Figure 1: Flow chart**


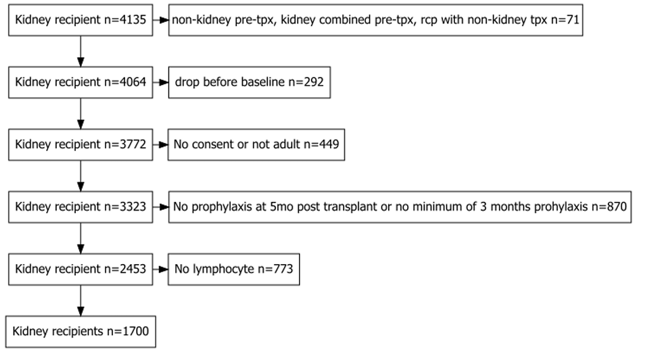


**Supplementary Table 1: Logistic regression model parameters to estimate the probability of a long antibiotic prophylaxis to derive inverse probability weights.**

|  | **Estimate** | **Standard error** | **P-value** |
| --- | --- | --- | --- |
| Age (years) | 0.006 | 0.005 | 0.25 |
| Not living donation | 0.210 | 0.135 | 0.12 |
| Use of anti-thymocyte globulin | 0.492 | 0.155 | <0.01 |
| Prior rejection | 0.885 | 0.148 | <0.01 |
| Cytomegalovirus infection within 7-month post-transplant | 0.329 | 0.138 | 0.02 |
| Absolute lymphocyte counts | 0.000 | 0 | 0.94 |
| Center 2 | -2.026 | 0.237 | <0.01 |
| Center 3 | -1.41 | 0.455 | <0.01 |
| Center 4 | -2.848 | 0.349 | <0.01 |
| Center 5 | -2.156 | 0.235 | <0.01 |
| Center 6 | -2.612 | 0.225 | <0.01 |

**Supplementary Table 2.** **Logistic regression model parameters of IPW model for the risk of bacterial** **infection**

|  | **Estimate** | **Standard error** | **P-value** |
| --- | --- | --- | --- |
| **Long TMP-SMX prophylaxis** | 0.21881 | 0.13067 | 0.094 |
| **Time since baseline (weeks)** |  |  |  |
| spline 1 | -0.02227 | 0.02192 | 0.310 |
| spline 2 | -0.00037 | 0.00077 | 0.628 |
| spline3 | 0.00001 | 0.00001 | 0.375 |
| **Age (years)** | 0.01089 | 0.00466 | 0.019 |
| **Not living donation** | 0.04806 | 0.12231 | 0.694 |
| **Use of anti-thymocyte globulin** | 0.40357 | 0.13489 | 0.003 |
| **Prior rejection** | 0.18983 | 0.13357 | 0.155 |
| **Cytomegalovirus infection within  7-month post-transplant** | 0.26586 | 0.12275 | 0.030 |
| **Lymphocyte counts (cells/mm3)** | -0.00004 | 0.00009 | 0.695 |
| **Center 2** | 0.01119 | 0.25037 | 0.964 |
| **Center 3** | -0.64817 | 0.62472 | 0.299 |
| **Center 4** | -0.01361 | 0.30109 | 0.964 |
| **Center 5** | 0.27278 | 0.23517 | 0.246 |
| **Center 6** | -0.11557 | 0.22806 | 0.612 |

TMP-SMX: Trimethoprim-sulfamethoxazole

**Supplementary table 3. Safety outcomes at 17-month follow-up by prophylaxis group**

| **Safety outcomes** | Short (n=1325) | Long (n=375) |
| --- | --- | --- |
| Glomerular filtration rate (ml/min/1.73m2), median (IQR) | 57 (45-72) | 51 (39-66) |
| Graft loss | 9 (0.7%) | 3 (0.8%) |
| Death | 12 (0.9%) | 9 (2.4%) |

| Type of opportunistic infections | Short (n=12) | Long (n=9) | Total (n=21) |
| --- | --- | --- | --- |
| Nocardia | 0 (0.0%) | 2 (22.2%) | 2 (9.5%) |
| Pneumocystis | 11 (91.7%) | 7 (77.8%) | 18 (85.7%) |
| Toxoplasmose | 1 (8.3%) | 0 (0.0%) | 1 (4.8%) |

**Supplementary table 4. Opportunistic infection (based on first episode), overall and by prophylaxis group.**
